# Can Metabolic Pathways Be Therapeutic Targets in Rheumatoid Arthritis?

**DOI:** 10.3390/jcm8050753

**Published:** 2019-05-27

**Authors:** Elsa Sanchez-Lopez, Anyan Cheng, Monica Guma

**Affiliations:** 1 Department of Pharmacology, UCSD School of Medicine, La Jolla, CA 92093-0663, USA; esl023@ucsd.edu; 2 Department of Medicine, UCSD School of Medicine, La Jolla, CA 92093-0663, USA; anc007@ucsd.edu

**Keywords:** synovium, metabolism, clinical trials, macrophages, fibroblasts

## Abstract

The metabolic rewiring of tumor cells and immune cells has been viewed as a promising source of novel drug targets. Many of the molecular pathways implicated in rheumatoid arthritis (RA) directly modify synovium metabolism and transform the resident cells, such as the fibroblast-like synoviocytes (FLS), and the synovial tissue macrophages (STM), toward an overproduction of enzymes, which degrade cartilage and bone, and cytokines, which promote immune cell infiltration. Recent studies have shown metabolic changes in stromal and immune cells from RA patients. Metabolic disruption in the synovium provide the opportunity to use in vivo metabolism-based imaging techniques for patient stratification and to monitor treatment response. In addition, these metabolic changes may be therapeutically targetable. Thus, resetting metabolism of the synovial membrane offers additional opportunities for disease modulation and restoration of homeostasis in RA. In fact, rheumatologists already use the antimetabolite methotrexate, a chemotherapy agent, for the treatment of patients with inflammatory arthritis. Metabolic targets that do not compromise systemic homeostasis or corresponding metabolic functions in normal cells could increase the drug armamentarium in rheumatic diseases for combination therapy independent of systemic immunosuppression. This article summarizes what is known about metabolism in synovial tissue cells and highlights chemotherapies that target metabolism as potential future therapeutic strategies for RA.

## 1. Introduction

Several recent reviews have highlighted metabolic changes in immunometabolism, stromal metabolism, and systemic metabolism in rheumatoid arthritis (RA) [1,2,3,4,5]. Qualitative changes to cellular metabolism are indeed essential to support physiological and pathological responses seen in the RA synovium. The phenotypic transformation of fibroblast-like synoviocytes (FLS) from quiescent cells to aggressive, metabolically active cells, the activation of synovial tissue macrophages (STM), and the recruitment of immune cells to the synovial tissue, all require an increased bioenergetic and biosynthetic demand. This is associated with changes in metabolism and energy production networks to support and enable rapid proliferation, migration, invasion, and proinflammatory mediator production in the hypoxic and nutrient deprived microenvironment that develops in the RA joint. We will focus in this review on clinical options for better stratification of patients through prognostic metabolomic analysis and on whether or not some of the therapeutic options explored in cancer could potentially increase the drug armamentarium in rheumatic diseases.

## 2. Joint Metabolism and Diagnostic Imaging

The use of metabolomic profiles to find novel biomarkers to help diagnose or stratify RA patients has been described [6]. Analysis of metabolites using one-dimensional nuclear magnetic resonance (1D NMR) spectroscopy or mass spectrometry coupled to gas or liquid phase separation techniques, have shown unique metabolic and lipid profiles in the plasma of RA patients and pre-symptomatic subjects compared with healthy donors [6]. For instance, acyl-carnitines, lysophosphatidylcholines (LPCs), and metabolites from tryptophan metabolism, were found to be enriched in plasma from pre-symptomatic patients [7]. This approach has highlighted urinary [8], serum, and synovial fluid metabolite signatures that distinguish RA from psoriatic arthritis and other diseases [6,9,10,11,12,13]. It has also described urine and plasma metabolic profiles that predict patient responses to biological therapies including etanercept, rituximab, and tocilizumab [14,15,16,17], highlighting the power of metabolomics in stratifying patients and directing RA treatment.

**Figure 1:**
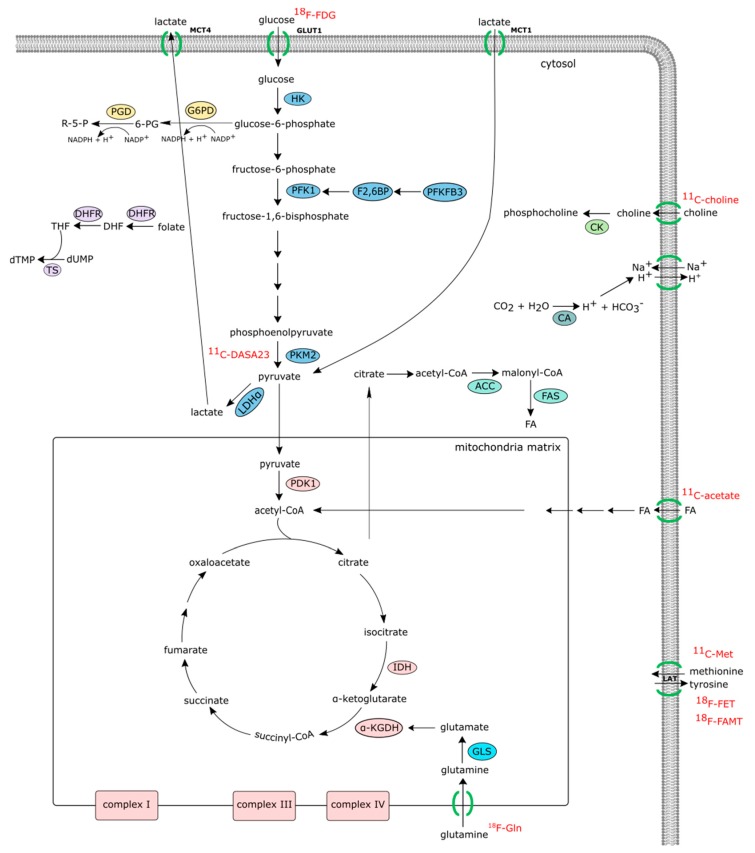
Positron emission tomography (PET) methods that provide information on the underlying biochemical processes. PET imaging not only can improve clinical diagnostics but also potentially predict treatment effects. ^18^F-FDG, 2-deoxy-2-(fluorine-18)fluoro-D-glucose, provides information on glycolysis and glucose uptake; ^11^C-DASA23, a class of N, N-diarylsulfonamides, is able to measure PKM2 uptake; ^18^F-Gln, ^18^F-(2*S*,4*R*)4-fluoroglutamine, allows for the monitoring of glutamine metabolism. ^11^C-Met, ^11^C-methionine; ^18^F-FET, O-(2-[^18^F]fluoroethyl)-L-tyrosine; ^18^F-FAMT, L-3-(18F)-Fluoro-α-methyl tyrosine; radiolabeled methionine and tyrosine can provide data on amino acid uptake and protein synthesis. Finally, ^11^C-acetate is converted to acetyl-CoA and used in mitochondria in TCA cycle or incorporated into cell membranes. MCT4, monocarboxylate transporter 4; GLUT1, glucose transporter 1; MCT1, monocarboxylate transporter 1; R-5-P, ribose-5-phosphate; PGD, phosphogluconate dehydrogenase; 6-PG, 6-phosphogluconate; G6PD, glucose-6-phosphate-dehydrogenase; HK, hexokinase; PFK1, phosphofructokinase 1; F2,6BP, fructose-2,6-bisphosphate; PFKFB3, 6-phosphofructo-2-kinase/fructose-2,6-bisphosphatase 3; dTMP, deoxythymidine monophosphate; dUMP, deoxyuridine monophosphate; TS, thymidylate synthase; THF, tetrahydrofolate; DHF, dihydrofolate; DHFR, dihydrofolate reductase; CK, choline kinase; PKM2, pyruvate kinase muscle isozyme M2; LDHα, lactate dehydrogenase A; CA, carbonic anhydrase; ACC, acetyl-CoA carboxylase; FAS, fatty acid synthase; PDK1, pyruvate dehydrogenase kinase 1; IDH, isocitrate dehydrogenase; α-KGDH, alpha-ketoglutarate dehydrogenase; GLS, glutaminase.

However, metabolomic profiles in serum, plasma, or urine do not necessarily correlate with joint metabolism. Other approaches are needed to identify synovial metabolic changes. Structural imaging techniques including radiography, ultrasound, and MRI, though very useful, fail to provide information on the underlying biochemical processes. Thus, non-invasive bioimaging techniques are of increasing interest to improve clinical diagnostics or to monitor arthritic disease. The ideal synovial biomarker probe would be a non-invasive probe able to identify cellular or molecular markers, which could help to discriminate at baseline between responders and non-responders to treatment, possibly leading to a more efficient and personalized treatment. Also, the analysis of serial synovial images would be particularly advantageous to detect changes in the synovial membrane so it can be used to determine the early effects of treatment. Thus, patient stratification based on pathological metabolic pathways prior to therapeutic intervention could be exploited in order to identify biomarker predictors of clinical outcomes and responses to therapy [18,19]. Noninvasive metabolic imaging modalities that include positron emission tomography (PET) and magnetic resonance spectroscopy (MRS) could help in patient stratification. Stable isotope resolved metabolomics studies of the synovium using liquid chromatography and gas chromatography mass spectrometry (LC-MS and GC-MS) can be utilized to complement noninvasive imaging techniques (Figure 1).

### 2.1. Positron Emission Tomography

PET imaging works by detection of gamma rays from positron emitting radionuclides that have been injected into the patient. The most commonly used radionuclide is ^18^F but there is a wide range of radionuclides available—the more commonly used isotopes include ^18^F, ^11^C, and ^15^O.

#### 2.1.1. ^18^F-FDG PET/CT (Positron Emission Tomography with 2-deoxy-2-(fluorine-18)fluoro-D-glucose Integrated with Computed Tomography)

^18^F-FDG works by entering the cell through glucose transporters where it is rapidly phosphorylated by hexokinase into ^18^F-FDG-6-phosphate where it can no longer be metabolized. The high consumption of glucose by advanced tumors made PET imaging with 18F-FDG an ideal probe to detect glycolytic tumors. However, the use of FDG to visualize tumor metabolic activity can also identify non-tumor cells that also have an increased metabolic activity at the inflammatory sites. In arthritis, synovial FLS and STM were shown to contribute to a high level of FDG-PET accumulation in the RA pannus [20]. Recent work has shown that the number of PET-positive joints in 28 and 68 joints was significantly correlated with the swollen and tender joint counts in RA patients [21]. ^18^F-FDG PET activity within days or weeks of initiating therapy correlates significantly with clinical endpoints. Thus, quantitative FDG-PET/CT-based assessment of inflammatory activity present in the joints of RA patients might be a promising approach for the whole-body assessment of RA disease activity and treatment response [22,23,24,25,26]. Additionally, it can also be used to detect high-risk disease complications at an early stage [27,28], such as atlanto-axial joint involvement, or co-morbidities including aortic inflammation [28]. A major drawback of PET imaging with ^18^F-FDG is that some normal cells in the brain, heart, and brown adipose tissue also have high metabolic rates and utilize above-average amounts of glucose, which often leads to the generation of false positive results.

#### 2.1.2. ^11^C-Choline PET/CT

Choline is a vitamin-like essential nutrient that is phosphorylated by choline kinase (ChoK), the enzyme that catalyzes the first step in the de novo synthesis of the phosphatidylcholine pathway [29]. Choline transporters CTL1 and CTL2 are expressed in the majority of the cells within the joint, in particular FLS and STM [30,31]. ^11^C-choline PET scanning, which is already in clinical use for identifying prostate cancer metastasis, showed increased choline uptake in inflammatory arthritis [32] and elevated levels of choline is present in RA FLS and synovium [33,34,35]. Although the use of choline radiotracers for diagnosis or stratification needs further examination, some studies have shown that there is a substantial difference in the synovial choline levels in RA patients compared to that of osteoarthritis (OA) patients, in particular in female RA patients [34].

#### 2.1.3. ^18^F-(2S,4R)4-fluoroglutamine Glutamine-, ^11^C-acetate-, ^11^C-methonine-PET/CT, O-(2-^18^Fluoroethyl)-L-tyrosine (FET), L-3-(^18^F)-Fluoro-α-methyl tyrosine (FAMT), and ^11^C-DASA23

Tumors or inflamed tissues do not solely rely on glucose. In recent years, the use of amino acid PET tracers is gaining attention for the diagnosis and evaluation of disease progression in several types of tumors, such as low-grade gliomas, and lung and breast cancer [36,37]. Some studies suggest that amino acid tracers are more sensitive in differentiating cancer cells from inflammatory cells, in part due to the upregulation of amino acid transport systems in cancer cells [38]. Transfer of amino acids across the plasma membrane is observed during metabolic stress. Several amino acid transporters have also been involved in the pathogenesis of other diseases including metabolic diseases such as obesity and diabetes [39]. Therefore, radiolabeling additional metabolites such as acetate, methionine, and glutamine with either ^18^F or ^11^C provide opportunities to perform broad profiling of tissue metabolism. For instance, it is believed that some tumors cannot be imaged with ^18^F-FDG as they do not derive their energy through glycolysis and instead probably use the glutaminolysis pathway as an alternate source of energy. Preliminary results suggest that ^18^F-(2*S*,4*R*)4-fluoroglutamine PET may be a new tool for probing in vivo metabolism of glutamine in cancer patients and for guiding glutamine targeted therapeutics [40,41]. ^11^C-acetate is converted to acetyl-CoA and used in mitochondria in the TCA cycle or incorporated into cell membranes. ^11^C-methionine and tyrosine tracers are used as a marker of amino acid uptake and protein synthesis primarily in cancer where uptake of the radiotracer correlates with tumor grade. Finally, Gambhir and colleagues reported the generation of a PET imaging probe specific for PKM2 using a class of N, N-diarylsulfonamides (DASA) known to promote PKM2 tetramer formation [42]. Although it was suggested that glutamine metabolism and glutaminase might be involved in the pathogenesis of RA fibroblasts [43], further studies are needed to determine whether or not these PET modalities could be useful for RA patient stratification.

### 2.2. Magnetic Resonance Spectroscopy Imaging (MRSI)

Magnetic Resonance (MR) imaging is most frequently used to determine anatomical registration of the tissues. Single-voxel MR spectroscopy (MRS) and multi-voxel MR spectroscopic imaging (MRSI) constitute another technique that enables detailed detection of cellular metabolic activity. The chemical composition forms a well-defined region of interest (ROI) or volume of interest in any organ of the human body that can be characterized using radiofrequency signals generated by nuclear spins of magnetic resonance active nuclei including ^1^H, ^31^P and ^13^C [44,45,46]. Most commonly, MRS has been used to evaluate endogenous ^1^H signals from choline-containing molecules, especially in the brain. ^31^P gathers information on the energy status of the tissue through the observation of various phosphate metabolites. Finally, ^13^C-labeled substrates provide dynamic metabolic flux information. However, one of the limitations of ^1^H MRS imaging is low sensitivity. Although several methods have allowed for an increase in the signal to noise ratio, this technology can mostly detect a few abundant metabolites in the imaged tissue, namely choline, glutamate, glutamine, lactate, aspartate, and phospholipid metabolites. Of interest, a few reports detected cerebral magnetic resonance spectroscopy changes in rheumatic diseases. Compared with healthy controls, patients with fibromyalgia had significantly higher levels of glutamate + glutamine and higher glutamate + glutamine/creatine (Glx/Cr) ratios in the posterior gyrus among other metabolic changes [47,48]. In another study, patients with active RA had a significantly higher ratio of choline to creatine and a significantly lower ratio of N-acetylaspartate to choline than did patients with inactive RA [49]. However, no studies have determined MRS changes in the arthritis synovium. As with other modalities of non-invasive imaging, monitoring response to therapy is one of the most promising aspects of MRS imaging.

### 2.3. Stable Isotope Resolved Metabolomics Studies

The noninvasive metabolism imaging methods discussed above can be complemented with mass spectrometry analysis of the inflamed synovial tissue. Little is known about metabolic or lipidomic profiling of the synovial tissue [34,35] although the increasing interest in synovial biopsies to obtain inflamed synovial tissue from joints [50] could improve understanding of the metabolic events in these diseases. It should be noted that although identification and quantification of endogenous and exogenous metabolic biomarkers can provide a metabolic snapshot of the status of a living organism, it cannot provide an unambiguous picture of the metabolic flux between different cellular compartments. For instance, an increase in the concentration of a metabolite can be associated with either the upregulation of the enzyme responsible for the metabolite synthesis, or the downregulation of the one consuming it. Several heavy isotopes including deuterium (^2^H), carbon (^13^C), nitrogen (^15^N), and oxygen (^18^O) have been used to aid in capturing the direction of a metabolic perturbation via the interpretation of stable isotope patterns and these datasets are nowadays increasingly being used in different mathematical modeling approaches, such as metabolic flux analysis. Stable isotope resolved metabolomics studies utilizes liquid chromatography mass spectrometry (LC-MS) or gas chromatography mass spectrometry (GC-MS) as a direct means to measure the distribution of labeled metabolites in the tissue and could potentially inform understanding of metabolic events in the RA synovium [51,52].

## 3. Metabolic Pathways as Therapeutic Targets in Rheumatoid Arthritis

Recent findings demonstrate the additional and consequent alterations in cellular signaling pathways and in the tumor microenvironment, including changes in the metabolism of glucose, lipids, and amino acids [53,54]. Therefore, in addition to ATP synthesis, metabolic changes appear to be a means of supplying cancer cells with the precursors of proteins, lipids, amino acids, and nucleic acids for building their cellular structure and maintaining their upregulated proliferation.

**Figure 2:**
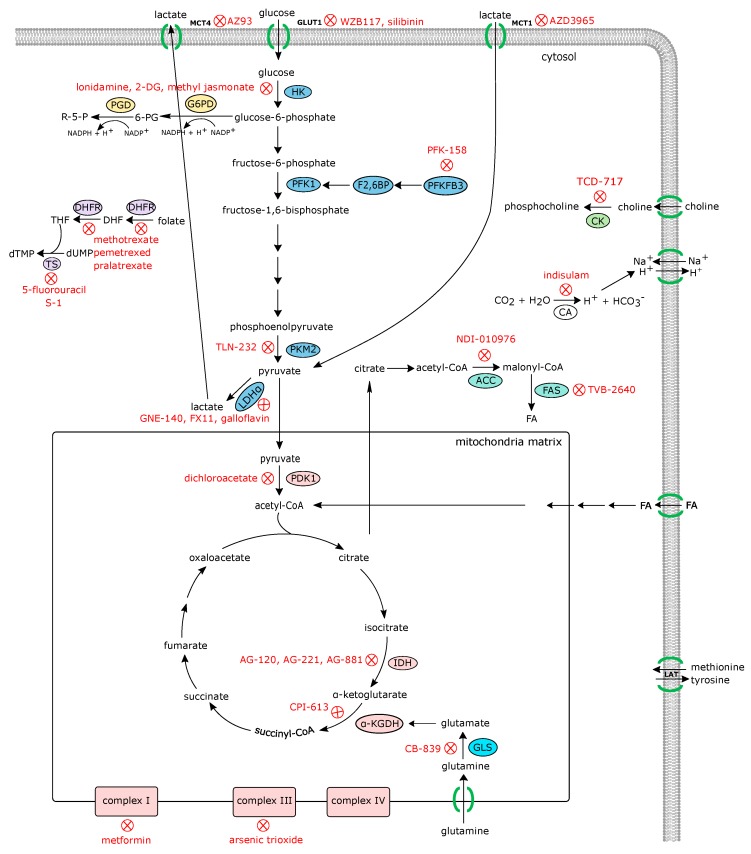
Anticancer agents targeting various metabolic pathways that are upregulated in activated cells. Since synovial tissue cells share many similar metabolic changes, these very same antimetabolites may also have potential uses in RA. MCT4, monocarboxylate transporter 4; GLUT1, glucose transporter 1; MCT1, monocarboxylate transporter 1; R-5-P, ribose-5-phosphate; PGD, phosphogluconate dehydrogenase; 6-PG, 6-phosphogluconate; G6PD, glucose-6-phosphate-dehydrogenase; HK, hexokinase; PFK1, phosphofructokinase 1; F2,6BP, fructose-2,6-bisphosphate; PFKFB3, 6-phosphofructo-2-kinase/fructose-2,6-bisphosphatase 3; dTMP, deoxythymidine monophosphate; dUMP, deoxyuridine monophosphate; TS, thymidylate synthase; THF, tetrahydrofolate; DHF, dihydrofolate; DHFR, dihydrofolate reductase; CK, choline kinase; PKM2, pyruvate kinase muscle isozyme M2; LDHα, lactate dehydrogenase A; CA, carbonic anhydrase; ACC, acetyl-CoA carboxylase; FAS, fatty acid synthase; PDK1, pyruvate dehydrogenase kinase 1; IDH, isocitrate dehydrogenase; α-KGDH, alpha-ketoglutarate dehydrogenase; GLS, glutaminase.

Due to the importance of metabolic alterations in the development and progression of cancer, several agents targeting cancer metabolism have been developed and evaluated under preclinical and clinical studies [55,56,57,58,59,60,61]. Some metabolism-targeting agents, such as mTOR inhibitors (rapamycin -sirolimus-, everolimus, and temsirolius) and metformin (an AMPK activator and mitochondrial Complex I inhibitor) are now approved for clinical use. Strategies targeting different metabolic alterations for anticancer therapy that could potentially be used in RA are detailed in the following sections and summarized in Figure 2, Table 1 and Table 2. In fact, rheumatologists already use the antimetabolites methotrexate (MTX) and leflunomide for the treatment of patients with inflammatory arthritis. Teriflunomide, the active metabolite of leflunomide, achieves its effects by inhibiting the mitochondrial enzyme dihydroorotate dehydrogenase (an enzyme involved in de novo pyrimidine synthesis). Methotrexate, developed as a folic acid analogue, inhibits purine and pyrimidine synthesis, although recent studies have indicated that other mechanisms such as adenosine accumulation can contribute to its effect in RA.

### 3.1. Glycolysis

The shift from oxidative phosphorylation to glycolytic ATP production is a common feature of activated and reactive cells such as cancer cells, fibroblasts, and macrophages [1,62,63]. As mentioned above, glucose uptake has been used to monitor tumor growth and to identify metabolically active sites such as RA joints or other inflammatory sites. Glycolysis is a multistep process that mobilizes glucose to produce pyruvate with a net yield of two adenosine triphosphate molecules. Although glycolysis is significantly less efficient producing ATP than the highly efficient mitochondria that generates 30-36 ATP molecules from a single glucose molecule, it is the preferential source of ATP under hypoxic conditions [64]. Inflammatory sites, such as inflamed joints, are overcrowded environments with scarce oxygen supply [65,66]. Synovial fluid is enriched in hypoxia-inducible factor 1 alpha (HIF1α), which contributes to RA pathogenesis, increases angiogenesis, inflammation, apoptosis, oxidative damage, and cartilage erosion [66]. Importantly, HIF1α is also a crucial regulator of glycolysis. In patients, synovial glycolytic marker expression positively correlated with reduced oxygen and macroscopic and microscopic changes in the joint, linking the glycolytic switch with hypoxia and inflammation [67]. The effect of HIF1α on glycolysis contributes to the pathogenic capacity of the majority of cells in the RA joint, including the production of proinflammatory cytokines such as IL-1β by macrophages [68], FLS survival [69], and FLS migration and invasion [66]. In addition, by inducing glycolysis, HIF1α serves as a metabolic checkpoint that supports Th17 development in detriment of Treg cells differentiation [70]. Yet, a recent report demonstrated a key function of HIF1α in driving the IL-10 expression in B cells [71].

Among the HIF1α−transcriptionally regulated genes, glucose transporter 1 (GLUT1) and lactate dehydrogenase (LDH) are upregulated in RA [1,60,72,73]. In the synovial lining, uptake of glucose is provided through Glut1 overexpression, which is accompanied by an increase in the glycolytic signature in the stromal compartment [60]. Glucose availability and uptake is crucial for the proliferative and invasive capacities of FLS [60]. The therapeutic use of GLUT1 blockers has been proposed to attenuate cancer cell proliferation [55], although none of the small molecules designed to block or reduce GLUT1 activity have met the standards to move forward to human studies. In contrast, a pilot study and Phase 1 study using an antisense oligonucleotide inhibitor of HIF1α has been tested in adults with advanced solid tumors, although the safety and efficacy reducing glycolysis is still unknown [74].

Another key glycolysis regulator downstream of HIF1α is the rate-limiting enzyme Hexokinase II (HK2), which is predominantly expressed in FLS within the RA joint [75]. Overexpression of HK2 in FLS provides a migratory and invasive advantage that is abolished when HK2 is ablated, and has attenuated the severity of bone and cartilage damage in a mouse model of inflammatory arthritis [75]. Importantly, ablation of glycolytic genes or treatment with 3-bromopyruvate, which antagonizes hexokinase II, significantly reduced the severity of mouse arthritis [60,76,77,78]. Methotrexate is a first-line therapeutic option for many RA patients. Interestingly, methotrexate treatment significantly reduced HK2 expression and glucose/fructose carriers (SLC2A5, a member of the solute carrier family 2) in human FLS, suggesting that FLS glycolytic activity can be modulated by methotrexate [79]. Although HK2 specific inhibitors are not available, steps downstream of HK2 can be inhibited by the use of 2-deoxyglucose (2-DG), which is a derivative of glucose that can be phosphorylated by HK2 but cannot be mobilized through the succeeding steps of glycolysis. This results in the accumulation of phosphorylated 2-DG causing product inhibition of HK2. Mouse studies have shown that 2-DG reduces cancer cell proliferation and the severity of the spontaneous murine models of arthritis [76]. These preclinical data have precipitated the examination of 2-DG in phase I/II trials for treatment of advanced cancer. Although the efficacy of 2-DG treatment in cancer progression is still unknown, only mild adverse effects have been observed, which include nausea and glucopenia, encouraging evaluation of this agent for treatment of inflammatory diseases such as RA, in which glycolysis is hyperactive.

Another important rate-limiting enzyme in glycolysis is pyruvate kinase, PKM2, which catalyzes phosphoenolpyruvic acid and ADP to pyruvate and ATP. PKM2 is overexpressed in many cancers and has a crucial role in glycolytic shift in immune cells [80]. PKM2 not only generates pyruvate, but also has multiple binding partners, such as HIF1α and Oct-4, which control inflammation and stem cell maintenance [81]. Monocytes/macrophages from patients with cardiovascular diseases, a common comorbidity of RA, have an increased glucose uptake and glycolytic flux, which causes mitochondrial stress and ROS production [82]. As a result, PKM2 dimerizes and translocates to the nucleus to activate the STAT3 transcriptional program that controls cytokine production [82]. Interestingly, inhibition of JAK/STAT3 signaling with Tofacinib, a drug approved for severe RA and active psoriasis, induces oxidative phosphorylation and maximal respiratory capacity of FLS while shutting down key glycolytic enzymes including HK2 and LDHA [83]. Currently, PKM2 inhibitor TLN-232/CAP-232 is evaluated in Phase II trials for refractory and metastatic renal cell carcinoma (NCT00422786) and recurrent metastatic melanoma (NCT00735332). It is reasonable to think that PKM2 inhibition may also attenuate the progression of the pathogenesis not only of RA but also of OA, as PKM2 overexpression controls glycolysis and extracellular matrix dynamic in chondrocytes [84]. Another critical enzyme in the glycolytic breakdown of glucose is the bifunctional 6-phosphofructo-2-kinase/fructose-2,6-biphosphatase (PFKFB) enzymes. In particular, PFKFB3 activity is defective in CD4+ T cells in RA patients which results in energy deprivation that prone cells to undergo apoptosis [85]. PFKFB3 inhibition reduced glucose uptake and utilization which resulted in decreased lactate production [86]. These inhibition of the glycolytic flux by small molecule inhibitors of PFKFB3 significantly reduces FLS migration and invasion, and the production of inflammatory mediators [67,86]. Conversely, fructose 1,6-bisphosphate (FBP), a high-energy intermediate of glycolysis, attenuated experimental arthritis by activating the anti-inflammatory adenosinergic pathway [87].

Active and sustained glycolytic activity also leads to lactate overproduction and release to the extracellular milieu. In tumors, lactate contributes to cell-to-cell communication and has an immunosuppressive effect on T cells [88]. Blockage of subtype-specific lactate transporters on T cells results in their release from the inflammatory site during peritonitis, supporting the role of lactate in T cell entrapment and function [89]. In RA patients, acidosis of synovial fluid occurs but it varies significantly between individuals. In obese mice, lactate dependent activation of HIF1α induces proinflammatory cytokine production [90]. Four members of the SLC16A family (SLC16A1, SLC16A3, SLC16A7 and SLC16A8), and two sodium-coupled lactate cotransporters (SLC5A12 and SLC5A8) are monocarboxylate transporters (MCTs) involved in lactate homeostasis [91,92]. Inhibition of MCTs or LDH activity by several non-specific inhibitors have been evaluated in preclinical cancer models; a specific inhibitor for human evaluation in patients remains to be discovered [93,94]. Lactate levels, or more precisely, the conversion of pyruvate into lactate, can be monitored by hyperpolarized MRS using ^13^C-pyruvate to assess tumor response [95]. As mentioned above, T cells in RA patients show deficient glycolytic flux, which results in low intracellular pyruvate levels and ATP scarcity. Metabolically challenged T cells initiate fatty acid synthesis and the formation of lipid droplets, which induces podosome scaffolding protein TKS5 overexpression, and results in hypermotility and T cell infiltration of synovial tissue [96].

Pyruvate molecules generated during glycolysis is converted to acetyl CoA to fuel the Krebs cycle. Emerging evidences show that Krebs cycle intermediates classically associated with metabolic functions also possess signaling functions as inflammatory mediators. Metabolic profiling has revealed itaconic acid as a potential marker of RA. Importantly, this increased levels of itaconic acid can be attenuated by treatment with infliximab, a biologic drug targeting tumor necrosis factor (TNF) [97]. In macrophages, itaconate acts as an anti-inflammatory factor that connect metabolism with oxidative and electrophilic stress responses and immune responses limiting HIF1α and cytokines production [98]. Succinate is another Krebs cycle intermediate that is abundant in RA synovial fluids. Synovial succinate correlates with enhanced release of IL-1β by macrophages in a mechanism that involves the overexpression of succinate receptor SUCNR1/GPR91 [99]. In addition, SUCNR1/GPR91 functions as a chemotactic signal for dendritic cells recruitment into lymph nodes which leads to Th17 cells expansion and the development of experimental antigen-induced arthritis [100]. Consistently, Sucnr1 ablation prevented articular hyperplasia, neutrophils infiltration, Th17 expansion, and the number of cytokines in the joint [100].

### 3.2. Glutaminolysis

Glutamine is another important carbon source that provides energy for respiration and serves as a precursor for the synthesis of nucleotides and proteins. Elevated glycolytic activity results in conversion of pyruvate to lactate rather than being incorporated into the tricarboxylic acid (TCA) cycle. To compensate, cancer cells rely on increased glutaminolysis [101]. Consistent with their metabolic similarities with cancer cells, reliance on glutamine is also a feature of FLS [43]. Inhibition or genetic ablation of glutaminase 1 (GLS1), the enzyme that converts glutamine to glutamate, inhibits RA-FLS proliferation and ameliorate the severity of experimental autoimmune arthritis [43]. Glutamate, which is converted to alpha ketoglutarate (a-KG) and channeled to the TCA cycle, is increased in the synovial fluid of RA patients and correlates with increased inflammation and IL-6 production by FLS [102]. In addition to RA, global and targeted metabolomic studies have shown that there is an enrichment of glutamine in the synovial fluid of OA patients compared to the synovial fluid from control individuals [103]. The use of radiotracers to visualize glutamine flux and metabolism in human tumors is under evaluation. ^18^F-(2S,4R)-4-fluoroglutamine has effectively depicted predominantly aggressive tumors of those which carried mutations related to glutamine metabolism [104]. Whether this technique can be used as an alternative to ^18^F-FDG or be used for RA diagnosis is still unknown. However, modulation of glutamine utilization under pathological conditions has been evaluated in cancer preclinical models. In particular, the blockage of glutamine transporter SLC1A5 has shown potent antitumor activity; however, its use in humans has been ruled out due to its effect on healthy cells [105]. Several small molecules that target GLS also have shown therapeutic potential for cancer patients [106,107]. The GLS1 inhibitor, CB839, which has proved to reduce cancer cells viability and proliferation, is currently being evaluated in clinical trials (NCT02071862, NCT02071888, NCT03428217).

### 3.3. Choline Metabolism

Increased phospholipid synthesis was classically associated with enhanced proliferation of cells. However, in recent years, several studies have revealed that phospholipid synthesis has more biological functions. In particular, the uptake, mobilization, and phosphorylation of choline by ChoK is critical for the de novo synthesis of the phosphatidylcholine pathway, also known as the Kennedy pathway. This pathway is elevated in activated cells and play important roles in inflammation [32,108,109,110]. RA FLS exhibit the so-called ‘GPC-to-PCho switch’ that is observed in cancer cells. Its activation is characterized by increased levels of phosphocholine (PCho) and total choline-containing metabolites along with a decrease in glycerophosphocholine (GPC)/PCho ratio, which indicates activation of this pathway [109]. FLS activators, including the proinflammatory cytokines TNF and IL-1β, and growth factors such as PDGF, induced ChoK accumulation [109]. Blocking ChoK activity by using a small molecule inhibitor limits the proliferative and migratory capacity of FLS by interfering with metalloproteases activity and Akt activation. In vivo, ChoK inhibition attenuated joint inflammation and destruction [109]. Of interest, a recent paper identified the choline transporters in RA FLS [31]. Their results suggested that CTL1 (high-affinity) and CTL2 (low-affinity), which were highly expressed in RA FLS, were critical for choline transport. They also showed that the choline uptake was significantly increased compared with that in OA FLS, suggesting an increase of this metabolism in RA FLS [31].

Activated macrophages also have a special avidity for choline, and choline transporters were also described in RA STM [30]. Inflammatory macrophages exhibit an enhanced uptake of choline that is rapidly phosphorylated by ChoK and mobilized through the Kennedy pathway to supply the phospholipids required for maintaining proper membrane fluidity and composition, facilitating cytokine production and release [111,112]. We have recently showed that when choline is limited, or after ChoK inhibition, the mitochondrial lipid profile is disrupted resulting in a reduction of ATP synthase activity and intracellular ATP, which subsequently activates the energy sensor and anti-inflammatory molecule AMPK [111]. AMPK activation then facilitates mitophagy and decreased NLRP3 inflammasome and IL-1β and IL-18 production [111].

Although the role of choline metabolism and choline kinase activity is well known in cancer cells, and many choline kinase inhibitors have shown antitumor properties, only TCD-717 has been evaluated in phase I clinical studies for the treatment of solid advanced tumors (NCT01215864). Importantly, the use of choline as a tracer, in addition to glucose, for cancer diagnosis and its potential use as a predictive factor of therapy response is currently on going in several trials. Although the safety of altering choline metabolism in humans still needs to be determined, further evaluation of choline metabolism and ChoK inhibition might open new approaches for controlling the progression of inflammatory diseases such as RA, OA, and gout.

### 3.4. Metabolic Regulators of Mitochondrial Function and Biogenesis (AMPK, mTOR, PGC1a)

Shift towards a glycolytic phenotype implies a reduction in mitochondrial function, which is also accompanied by impaired mitochondrial biogenesis. Reduced cellular ATP triggers the activation of the energy sensor AMPK, which then phosphorylates multiple downstream targets and turns off biosynthetic pathways that consume ATP, including the synthesis of protein, fatty acids, and lipids, while facilitating glucose uptake and fatty acid oxidation to promote ATP production [113]. AMPK also is known for triggering autophagy/mitophagy through phosphorylation of ULK1 and MFF, a process that saves energy by reutilizing waste or unused materials [114,115]. AMPK is essential for RA T cells. AMPK activation is dependent on myristoylation, and in RA it has been shown that T cells aberrant N-myristoyltranferase prevented AMPK activation. This induced a mTORC1 overactivation that facilitated the differentiation of Th1 into Th17 cells [116]. Furthermore, pharmacological activation of AMPK using a specific AMPK agonist reduced the expression and release of IL-6 [117]. Importantly, methotrexate is able to induce the activation of AMPK, which correlates with reduced inflammatory response in macrophages stimulated with LPS and TNF [118]. Metformin and its analog phenformin are glucose-lowering drugs used for diabetes mellitus patients. Although their exact mechanism of action is not clearly understood, their well-known effect on AMPK activity may also contribute to the beneficial secondary effects of these drugs, as reducing inflammatory markers, improving lipid metabolism, and attenuating experimental autoimmune arthritis [119,120,121,122,123,124]. Its potential role as immune modulator indicate that treatment with metformin may be of special interest in inflammatory and autoimmune diseases [125]. In particular, several clinical trials are evaluating the role of metformin in RA and co-morbidities (NCT02246257, NCT03686657), and in psoriatic arthritis (NCT02188654).

AMPK activation is known to reduce mTOR signaling and activate autophagy. The mTOR signaling pathway, which is a key regulator of protein synthesis, is unusually active in many cancers and autoinflammatory and autoimmune diseases. mTOR signaling is also crucial for monocyte differentiation from myeloid progenitors [126]. Inhibition of mTOR can be achieved by rapamycin, also known as sirolimus, or its analogs, so-called rapalogs, that are FDA approved to treat advanced cancer [127]. Currently, the use of rapalogs is under evaluation in several clinical trials to determine their possible use in autoimmune diseases. The small molecule temsirolimus was evaluated in active RA patients, concomitant with methotrexate (NCT00076206), and rapamycin was also assessed by pharmacodynamic studies on hyperuricemia in gout patients (NCT02959918). In addition, a recently completed trial evaluating the effect of sirolimus in pediatric autoimmune diseases, including systemic lupus erythematosus (SLE), inflammatory bowel disease, and RA showed that sirolimus led to a complete and long-lasting response in the majority of pediatric patients, suggesting that sirolimus should be considered in the treatment of children with SLE [128].

Another important regulator of multiple cellular processes that is downregulated in RA is SIRT1. SIRT1 has an important role in suppressing the activation of NF-κB-dependent inflammatory responses including COX-2 and iNOS production, and promotes the activation of antioxidant transcriptional program [129]. Both SIRT1 and SIRT6 are essential for maintaining cartilage homeostasis and the use of SIRT1 activating compounds has been proposed as therapeutic approach in OA [130]. In this sense, resveratrol, a polyphenol found in wines, is extensively studied as an SIRT activator, and exhibit potent antioxidant, anti-inflammatory, and anticancer activities [131]. Recently, resveratrol was reported to suppress the severity of inflammatory arthritis in mice [132,133]. A randomized controlled clinical trial of 100 RA patients (68 female, 32 male) showed that the clinical markers and the disease activity score assessment was lowered by resveratrol [134]. More importantly, serum biochemical markers, such as C-reactive protein, erythrocyte sedimentation rate, undercarboxylated osteocalcin, matrix metalloproteinase-3, TNF, and IL-6 were also significantly decreased in resveratrol-treated patients [134]. In another study, treatment with resveratrol as an adjuvant with meloxicam improved pain and joint function [135]. The evaluation of resveratrol in knee OA is also currently under evaluation (NCT02905799).

### 3.5. Amino Acid Uptake

Increased amino acid uptake is often found in certain types of tumors to fuel metabolism and protein synthesis [37]. Besides glutamine, other amino acids, including serine, have been shown to be important for initiating proper inflammatory responses. In macrophages, serine metabolism to produce glutathione is required for transcriptional regulation of IL-1β [136]. In proinflammatory macrophages, influx of the branch-chain amino acid leucine also contributes to cytokine production via mTORC1-induced glycolytic reprogramming [137]. Consistently, branched-chain aminotransferase 1 (BCAT1) controls oxygen consumption and glycolysis in macrophages. Its inhibition by ERG240 limits infiltration of inflammatory macrophages and therefore reduces the severity of experimental inflammatory arthritis [138]. In addition, RA synovium is enriched in L-type amino acid transporter gene LAT1 [139]. Expression of SLC7A5/LAT1 was found to be significantly elevated in monocytes derived from patients with RA [137]. RA FLS also overexpress LAT1 and has an increased uptake of leucine after IL-17 stimulation, which potentiates the FLS migratory capacity that was eliminated by blocking LAT1 [139]. Thus, tracing and targeting the uptake of serine and leucine may be useful to determine and decrease the inflammatory status in RA joints.

Another important amino acid for macrophage function is arginine. Arginine is the substrate of two enzymes, arginase to produce ornithine and urea, and nitric oxide synthase (NOS) to generate citrulline and nitric oxide (NO). Macrophages couple the arginine metabolism with polarization and functional phenotype [140,141]. Inflammatory macrophages, classically called M1, preferentially overexpress NOS and use arginine for NO production, which is a key effector of microbicidal activity [141]. In contrast, M2 macrophages preferentially express arginase to generate ornithine, which is the precursor of polyamines that contributes to proliferation and restoration of tissue homeostasis [141]. Of interest, arginase protein and activity are elevated in serum from RA patients [142]. Inhibition of arginase by different approaches has been clinically assessed in human cancers.

In the past few years, the enzyme indoleamine 2,3-dioxygenase (IDO), involved in the metabolism of the essential amino acid tryptophan, has gained attention. IDO is overexpressed in cancer and mediates immune tolerance. T cells exhibit sensitivity to tryptophan deprivation and to kynurenines, the products of tryptophan degradation [143,144]. IDO inhibitors were well tolerated in phase I studies [145,146]. As IDO inhibitors boosted “immunogenic” chemotherapy or immune checkpoint drugs [147], current clinical trials focus on evaluating the effects of combining IDO inhibitors with taxotere, sipuleucel-T (dendritic cell vaccine), and anti-PD1 (NCT03047928, NCT01219348, NCT01982487). Although increased tryptophan degradation measured as kynurenines/tryptophan ratio is elevated in the blood of RA patients [148], the performance of IDO inhibitors in arthritis is under debate as some works showed that IDO inhibition exacerbated disease severity in mouse models of arthritis [149,150].

### 3.6. Fatty Acid Synthesis

Fatty acid metabolism is a dynamic process of anabolic and catabolic reactions to maintain energy homeostasis. Synthesis of fatty acids is essential for building up metabolic intermediates to store energy, maintain cell membrane structures, and participate in intracellular and intercellular communication. When energy is needed, the cell can break down the fatty acids through β-oxidation. Balance between synthesis and degradation is impaired in many diseases, which leads to lipid accumulation. Fatty acid synthase, FASN, a key enzyme in the de novo synthesis of lipids, is found to be overexpressed in many cancers [151,152,153]. In macrophages, FASN is essential for maintaining optimal membrane composition [154]. Deletion of FASN in macrophages impairs the retention of cholesterol in the plasma membrane and alters Rho GTPase-dependent cell adhesion and migration [154]. Inhibition of FASN is under evaluation in several clinical trials in cancer patients (NCT03179904, NCT02980029, NCT02595372), after promising preclinical studies in animal models [155].

Fatty acids elongation determines chain length of saturated, monosaturated, and polyunsaturated fatty acids in cellular lipids. In some cancers, the enzymes that ultimately control the elongation of the fatty acids, the fatty acid elongases (ELOVL1-7), are dysregulated and can be used as predictive factors [156,157,158]. The role of fatty acid synthesis and elongation in the behavioral changes of RA FLS is yet to be clarified. FLS have a potent migratory and invasive capability, which presumably will require an active lipid remodeling. In addition, many of the inflammatory molecules that are present in the synovium stimulate fatty acid synthesis. Evaluation of this biosynthetic pathway, not only in FLS but also in other cells that play a role in the arthritic joint, such as macrophages, may offer novel targets to attenuate joint damage.

## 4. Conclusions

In the last decade, the use of new technologies to conduct metabolomic and lipidomic studies in body fluids have provided new insights into immune-mediated inflammatory diseases such as RA. It now become clearer that alterations in the lipid profile and the hyperactivation of metabolic pathways are hallmarks of RA, and that they can be potential biomarkers and therapeutic targets. Findings in human synovium or peripheral blood and preclinical studies in mouse models of inflammatory arthritis strongly suggest that agents that interfere with lipid metabolism and certain steps of glycolysis or other energy-related pathways can be therapeutic in RA (Table 3). Yet, we must emphasize that we are far from understanding the pathways that discern normal from pathogenic metabolic phenotypes of synovial cells. The widespread use of imaging techniques or mass spectrometry analysis in larger and more heterogeneous cohorts can provide signatures of metabolic disruption under pathogenic conditions. Additionally, further efforts are needed to fine tune metabolism by designing or improving current medications, or by using prodrugs that can only be activated when the target is hyperactive, to ensure the successful use of metabolic drugs with minimal off-targets. Whether or not chemotherapies that modulate the metabolism truly present an option to increase the drug armamentarium in rheumatic diseases remains to be determined.

## Figures and Tables

**Table 1:** Clinical trials of drugs that target various steps of the glycolytic and mitochondrial metabolic pathways.

Drug	Pathway	Disease	Trial Status	Identifier #
Silibinin	glycolysis (glut1)	liver cancer	phase I	NCT01129570
		prostate cancer	phase II	NCT02146118
		hypertension	phase IV	NCT03538327
RO7070179	HIF1α	Hepatocellular carcinoma	Phase I	NCT02564614
lonidamine	glycolysis (HK)	enlarged prostate	phase III	NCT00435448
2-DG	glycolysis (HK)	advanced solid tumor	phase I	NCT00096707
		prostate cancer	phase I/II	NCT00633087
PFK-158	glycolysis (PFKFB3)	advanced solid tumors	phase I	NCT02044861
TLN-232	glycolysis (PKM2)	melanoma	phase II	NCT00735332
		renal cell carcinoma		NCT00422786
AZD3965	lactate uptake (MCT 1)	advanced solid tumor	phase I	NCT01791595
Indisulam	H^+^ secretion	gastric cancer	phase I/II	NCT00165594
		kidney cancer	phase II	NCT00059735
Dichloroacetate	PDK1	head and neck cancer	phase I	NCT01163487
		breast, lung cancer	phase II	NCT01029925
CPI-613	aKGDH	small cell lung cancer	phase I	NCT01931787
		lymphoma, leukemia	phase II	NCT03793140
AG-120	isocitrate DH	advanced solid tumor	phase I	NCT02073994
		leukemia	phase II	NCT03503409
		cholangiocarcinoma	phase III	NCT02989857
AG-221	isocitrate DH	leukemia	phase I	NCT03728335
		leukemia	phase II	NCT03744390
		advanced solid tumor	phase I/II	NCT02273739
AG-881	isocitrate DH	glioma	phase I	NCT02481154
metformin	Mitochondrial complex I	RA	phase I/II	NCT03686657
		prostate cancer	phase II	NCT03137186
		SLE	phase IV	NCT02741960
arsenic trioxide	Mitochondrial complex III	leukemia	phase II	NCT03624270

glut1, glucose transporter 1; HK, hexokinase; PFKFB3, 6-phosphofructo-2-kinase/fructose-2,6-bisphosphatase 3; MCT1, monocarboxylate transporter 1; MCT4, monocarboxylate transporter 4; PDK1, pyruvate dehydrogenase kinase 1; α-KGDH, alpha-ketoglutarate dehydrogenase; isocitrate DH, isocitrate dehydrogenase.

**Table 2:** Clinical trials of drugs that target other metabolic pathways than glycolysis, involved in the upregulated synthesis, proliferation, and survival of cells that have undergone metabolic rewiring.

Drug	Pathway	Disease	Trial Status	Identifier
CB-839	glutaminase	advanced solid tumor	phase I	NCT02071862
		renal cell carcinoma	phase II	NCT03428217
ADI-PEG20	arginine availability	breast cancer	phase I	NCT01948843
		hepatocellular cancer	phase II	NCT00056992
		hepatocellular cancer	phase III	NCT01287585
TVB-2640	fatty acid synthase	advanced solid tumor	phase I	NCT02223247
		NSCLC	phase II	NCT03808558
NDI-010976	acetyl-CoA carboxylase	healthy obese adults	phase I	NCT02876796
TCD-717	choline kinase	advanced solid tumor	phase I	NCT01215864
epacadostat	indoleamine-2,3-dioxygenase	solid tumor	phase I	NCT03471286
		MDS	phase II	NCT01822691
indoximod	indoleamine-2,3-dioxygenase	prostate cancer	phase II	NCT01560923
rapamycin	mTOR	thyroid cancer	phase II	NCT00936858
everolimus	mTOR	prostate cancer	phase II	NCT00976755
		kidney cancer	phase III	NCT01120249
temsirolimus	mTOR	RA	phase II	NCT00076206
leflunomide	Pyrimidine synthetase		approved	
methotrexate	dihydrofolate reductase		approved	
pemetrexed	dihydrofolate reductase		approved	
pralatrexate	dihydrofolate reductase		approved	
5-fluorouracil	thymidylate synthase		approved	
S-1	thymidylate synthase		approved	
pentostatin	adenosine deaminase		approved	
6-mercaptopurine	adenine deaminase		approved	
azathioprine	purine synthesis		approved	
cladribine	adenosine deaminase		approved	
gemcitabine	ribonucleotide reductase		approved	
cytarabine	DNA polymerase/ribonucleotide reductase		approved	
fludarabine	DNA polymerase/ribonucleotide reductase		approved	
hydroxyurea	ribonucleotide reductase		approved	

mTOR, mammalian target of rapamycin; NSCLC, non-small cell lung cancer; MDS, myelodysplasic syndrome.

**Table 3:** Preclinical data of drugs that target metabolic pathway.

Pathway	Animal Model	Effect on Cells	Reference
glycolysis (HK II)	K/BxN	Genetic ablation of HK2 inhibits invasive capacities of FLS and secretion of inflammatory ctytokines.	[75,78],
glycolytic inhibitors (2DG, bromopyruvate and ionidamine)	K/BxN, CIA, SKG	Glycolytic inhibitors reduced FLS aggressive phenotype, decrease effector CD4+ cells, and modulated Th17/Treg differentiation.	[60,76,77,78]
glycolysis (PFKFB)	CIA	PFKFB3 inhibition reduced FLS migration and invasion, and the production of inflammatory mediators	[86]
glycolysis (FPB)	AIA, zymosan	Systemic generation of extracellular adenosine and subsequent activation of adenosine receptor A2a	[87]
succinate receptor (SUCNR1)	AIA	Sucnr1 guides dendritic cells into the lymph nodes, leading the expansion of the Th17-cell population	[100]
glutaminase 1 (GLS1)	K/BxN	Inhibition or genetic ablation of glutaminase 1 (GLS1) inhibits RA-FLS proliferation	[43]
choline kinase	K/BxN	Blocking choline kinase activity limits the proliferative and migratory capacity of FLS	[109]
amino acid uptake (BCAT1) metformin	CIA CIA, CAIA, K/BxN	BCAT Inhibition reduces IRG1 and itaconate levels in macrophages.	[138]
		Metformin modulated Th17/Treg differentiation and osteoclastogenesis.	[122,123,124]

CIA: collagen-induced arthritis, AIA: antigen-induced arthritis, CAIA: collagen antibody-induced arthritis.
